# Cytokine Profiles of Non-Small Cell Lung Cancer Patients Treated with Concurrent Chemoradiotherapy with Regards to Radiation Pneumonitis Severity

**DOI:** 10.3390/jcm10040699

**Published:** 2021-02-11

**Authors:** Bae Kwon Jeong, Jin Hyun Kim, Myeong Hee Jung, Ki Mun Kang, Yun Hee Lee

**Affiliations:** 1 Department of Radiation Oncology, Gyeongsang National University School of Medicine and Gyeongsang National University Hospital, Jinju 52727, Korea; blue129j@hamail.net; 2 Institute of Health Sciences, Gyeongsang National University, Jinju 52757, Korea; ajini7044@hanmail.net (J.H.K.); jsk92@naver.com (K.M.K.); 3 Biomedical Research Institute, Gyeongsang National University Hospital, Jinju 52757, Korea; yallang7@daum.net; 4 Department of Radiation Oncology, Gyeongsang National University School of Medicine and Gyeongsang National University Changwon Hospital, Changwon 51472, Korea; 5 Department of Radiation Oncology, Bucheon St. Mary’s Hospital, College of Medicine, The Catholic University of Korea, Bucheon 14647, Korea

**Keywords:** cytokine, non-small cell lung cancer, chemoradiotherapy, radiation pneumonitis

## Abstract

The immunologic aspects of radiation pneumonitis (RP) are unclear. We analyzed variations in cytokine profiles between patients with grade (Gr) 0–1 and Gr ≥ 2 RP. Fifteen patients undergoing concurrent chemoradiotherapy for non-small cell lung cancer were included. Blood samples of 9 patients with Gr 0–1 and 6 with Gr ≥ 2 RP were obtained from the Biobank. Cytokine levels were evaluated using an enzyme linked immunosorbent assay at before radiotherapy (RT) initiation, 1, 3, and 6 weeks post-RT initiation, and 1 month post-RT completion. Concentrations of granulocyte colony-stimulating factor (G-CSF), interleukin (IL)-6, IL-10, IL-13, IL-17, interferon (IFN)-γ, tumor necrosis factor (TNF)-α, and transforming growth factor (TGF)-β were analyzed; none were related to the occurrence of Gr ≥ 2 RP at pre-RT initiation. At 3 weeks, relative changes in the G-CSF, IL-6, and IFN-γ levels differed significantly between the groups (*p* = 0.026, 0.05 and 0.026, respectively). One month post-RT completion, relative changes of IL-17 showed significant differences (*p* = 0.045); however, relative changes in TNF-α, IL-10, IL-13, and TGF-β, did not differ significantly. Evaluation of changes in IL-6, G-CSF, and IFN-γ at 3 weeks after RT initiation can identify patients pre-disposed to severe RP. The mechanism of variation in cytokine levels in relation to RP severity warrants further investigation.

## 1. Introduction

The lung is a radiation-sensitive organ, and 50–90% of patients receiving radiation therapy (RT) for lung cancer develop radiation pneumonitis (RP) one to six months after RT completion. Among them, 20–30% progress to Grade (Gr) ≥ 2 radiation pneumonitis (RP) [1]. Factors associated with Gr ≥ 2 RP include radiation therapy dosimetric parameters and patient- and disease-related clinical factors; among these, dosimetric factors are known to be the most important [2,3]. However, despite being treated according to the dose constraint guidelines for radiation treatment planning, patients still experience Gr ≥ 2 RP. It is believed that the underlying mechanism associated with the patient’s robust immune reaction may be associated with this [4].

Little is known regarding the immunological mechanisms involved in radiation lung damage. Radiation lung damage occurs over complex stages, involving lung epithelial cells, endothelial cells, and immune cells, and their interaction with various pro-inflammatory cytokines and chemokines [5]. Transforming growth factor (TGF)-β, interleukin (IL)-1, and IL-6 levels have been shown to be related to the development radiation pneumonitis. However, the results vary among researchers, making it difficult to interpret [6].

The study of combination treatment of immunotherapy and radiation therapy is still in its early stages, and its effects and side effects are not well known. In unresectable stage III non-small lung cancer (NSCLC), durvalumab consolidation therapy after concurrent chemoradiotherapy (CCRT) has been performed as a standard treatment since the phase 3 PACIFIC trial [7]. Although progression-free survival (PFS) has improved owing to immunotherapy, there have been concerns regarding an increase in the incidence of severe RP. Several recent studies have reported that the incidence of RP increases after the addition of durvalumab, and that there are differences in the timing and pattern of its occurrence [8]. By knowing the immunological mechanism of radiation lung damage and identifying early positive markers, serious side effects can be prevented; this will also contribute to improving treatment efficacy.

In this study, we evaluated the cytokine profiles of NSCLC patients treated with CCRT. The possible differences in the cytokine profiles between patients with Gr 0–1 RP and Gr ≥ 2 RP were analyzed.

## 2. Materials and Methods

### 2.1. Patients

This study was approved by the institutional review board (IRB) of the Gyeongsang National University Hospital [GNUH 2017-10-014-001]. The inclusion criteria were as follows: (1) patients had been treated with CCRT for NSCLC; (2) treatment was delivered with a radical aim; (3) total radiation dose delivered was more than 50 Gy; (4) the follow up was of a minimum of 3 months after treatment; (5) computed tomography or chest radiograph data were available for RP evaluation; and (6) blood samples were available before and during RT and 1 month after RT completion. The blood samples of the patients treated with CCRT for NSCLC were provided by the Gyeongsang National University Hospital Human Biobank. Patients’ clinical data, including those related to age, sex, comorbidities, smoking history, cancer stage, and histology were obtained from the Biobank. Treatment information, including total radiation dose, RT start and finish date, and chemotherapy regimen were also obtained. The severity of RP was graded according to the Common Terminology Criteria for Adverse Events (CTCAE) version 4.0.

### 2.2. Radiation Therapy

The computed tomography simulation was performed using a vac-lock device. The gross tumor volume was defined as the primary lung mass volume plus metastatic lymph node volumes in any imaging study. The clinical target volume was delineated to include a 5–10 mm margin around the gross tumor volume, ipsilateral hilum, and adjacent lymphatic chains. The planning target volume margin was generated according to lung tumor movement on two-dimensional fluoroscopy. Radiation treatment was planned using the eclipse planning system (Varian Medical Systems, Palo Alto, CA, USA) in accordance with institutional dose constraint guidelines. For the total lung, mean lung dose was constrained to less than 20 Gy; V_20_ was less than 30–36%, and V_5_ was less than 60%. The maximum dose to the spinal cord was less than 45 Gy. For the heart, V_30_ was constrained to less than 45%, and the mean dose was less than 26 Gy. Both, 3-dimensional conformal RT (3D-CRT) and intensity-modulated RT (IMRT) were allowed.

### 2.3. Blood Samples

Blood samples were stored at 4 °C before obtaining the plasma. The samples were then centrifuged for 10 min at 2000× *g*, 4 °C. The plasma collected were then immediately stored at −70 °C in the Biobank (Biobank, Gyeongsang National University Hospital, a member of Korea Biobank Network). Specimen collection, centrifugation, and storage were all performed by the Biobank. Blood samples from 9 patients with Gr 0–1 RP and 6 patients with Gr ≥ 2 RP were used. Samples were collected before RT initiation, 1, 3, and 6 weeks after RT initiation, and 1 month after RT completion. To harvest the plasma of each patient, blood was collected in heparin vacutainers and centrifuged at 1200 rpm for 10 min.

### 2.4. Cytokines Analysis

Enzyme linked immunosorbent assay was performed to analyze each circulating cytokine. Granulocyte colony-stimulating factor (G-CSF), IL-6, IL-10, IL-13, IL-17, interferon-γ (IFN-γ), tumor necrosis factor-α (TNF-α), and TGF-β were included. Each cytokine level was measured using a Quantikine^®^ ELISA (enzyme-linked immunosorbent assay) kit (R&D Systems, Minneapolis, MN, USA) according to the manufacturer’s instructions. Calibration of each test was based on the standard curve. The density of color was proportional to the amount of cytokine present in the well.

### 2.5. Statistical Analysis

Descriptive statistics were used to show the patient and treatment characteristics and cytokine levels at each time point. Mann–Whitney U and Fisher’s exact tests were performed to identify the significant clinical factors related to Gr ≥ 2 RP. The Mann–Whitney U test was performed to compare the cytokine levels at each time point between patients with Gr 0–1 RP and Gr ≥ 2 RP. Relative changes at each time point were defined as follows: for 1 week: (cytokine levels at 1 week—cytokine levels at pre-RT)/cytokine levels at pre-RT. A linear mixed model was used to compare the differences according to time or groups. The SPSS version 20 software (IBM Corp., Armonk, NY, USA) was used for statistical analyses. A *p*-value of ≤0.05 was considered significant.

## 3. Results

### 3.1. Patients and Treatment Characteristics

A total of 15 patients with NSCLC were included. Table 1 shows the characteristics of the patients and their treatments. Six and 9 patients who had stage IIIA and stage IIIB disease, respectively, were included. Radiation was delivered to a median dose of 59.4 Gy (range, 55.8–66 Gy) at 1.8–2 Gy per fraction. The regimens of CCRT included paclitaxel-cisplatin (*n* = 7 patients) and paclitaxel-carboplatin (*n* = 8 patients). A total of 9 patients demonstrated Gr 0–1 RP and 6 patients had Gr ≥ 2 RP. Sex, age, smoking history, presence of underling lung disease, clinical stage, histology, total RT dose, and RT techniques were not related to the Gr ≥ 2 RP (Table 1).

### 3.2. Cytokine Levels at Pre-Radiation Therapy (RT), during, and after Radiotherapy

**Figure 1:**
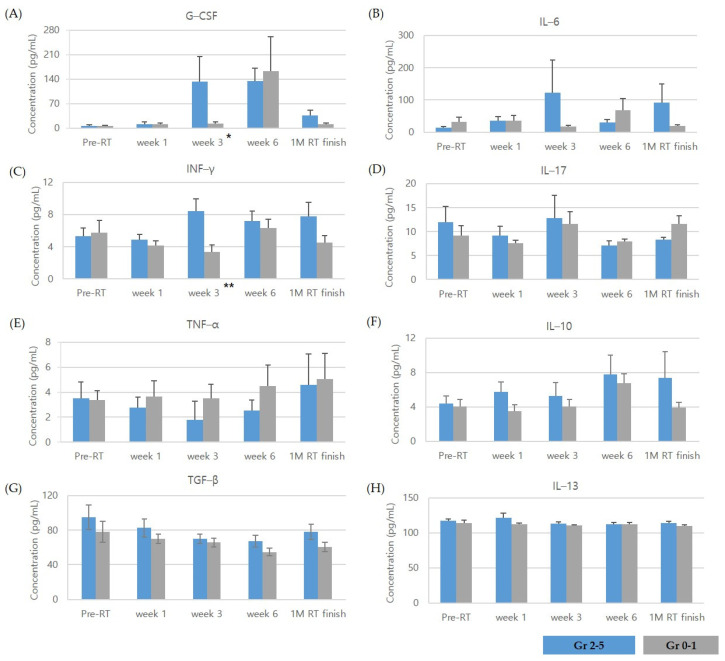
Cytokine levels at pre-RT, during, and after radiotherapy in patients with Grade ≥ 2 and Grade 0–1 radiation pneumonitis: (**A**) granulocyte colony-stimulating factor (G-CSF); (**B**) interleukin (IL)-6; (**C**) interferon (INF)-γ; (**D**) IL-17; (**E**) tumor necrosis factor-α (TNF-α); (**F**) IL-10; (**G**) transforming growth factor-β (TGF-β); (**H**) IL-13 levels. Data are expressed as the mean ± standard error (SE). * *p* = 0.008, ** *p* = 0.001.

The cytokine levels at pre-RT, 1, 3, and 6 weeks after RT initiation, and 1 month after RT completion are presented in Figure 1.

None of the cytokine levels at pre-RT were related to Gr ≥ 2 RP occurrence. For the G-CSF levels, the Gr ≥ 2 RP group had its peak concentration at 3 weeks after RT initiation, while the Gr 0–1 group had its peak at 6 weeks after RT initiation. The cytokine levels at 3 weeks were significantly different between the 2 groups (*p* = 0.008). IL-6 levels of the Gr ≥ 2 RP group demonstrated peak concentration at 3 weeks after RT initiation. However, those levels were not significantly higher compared to those of the Gr 0–1 group (*p* = 0.282). IFN-γ levels were significantly higher in the Gr ≥ 2 RP group at 3 weeks after RT initiation as compared to the Gr 0–1 group (*p* = 0.001). As for the other cytokines, their levels did not show any significant difference between 2 groups before, during, and after RT. We analyzed whether the changes in the cytokines before, during and, after the CCRT were different between the two groups. For IL-17, the changes over time showed a significant difference between patients with Gr 0–1 RP and Gr ≥ 2 RP (*p* = 0.017). For the other cytokines, the pattern of temporal changes between the two groups did not show any significant difference.

### 3.3. Relative Changes Compared to Pre-RT Level

**Figure 2:**
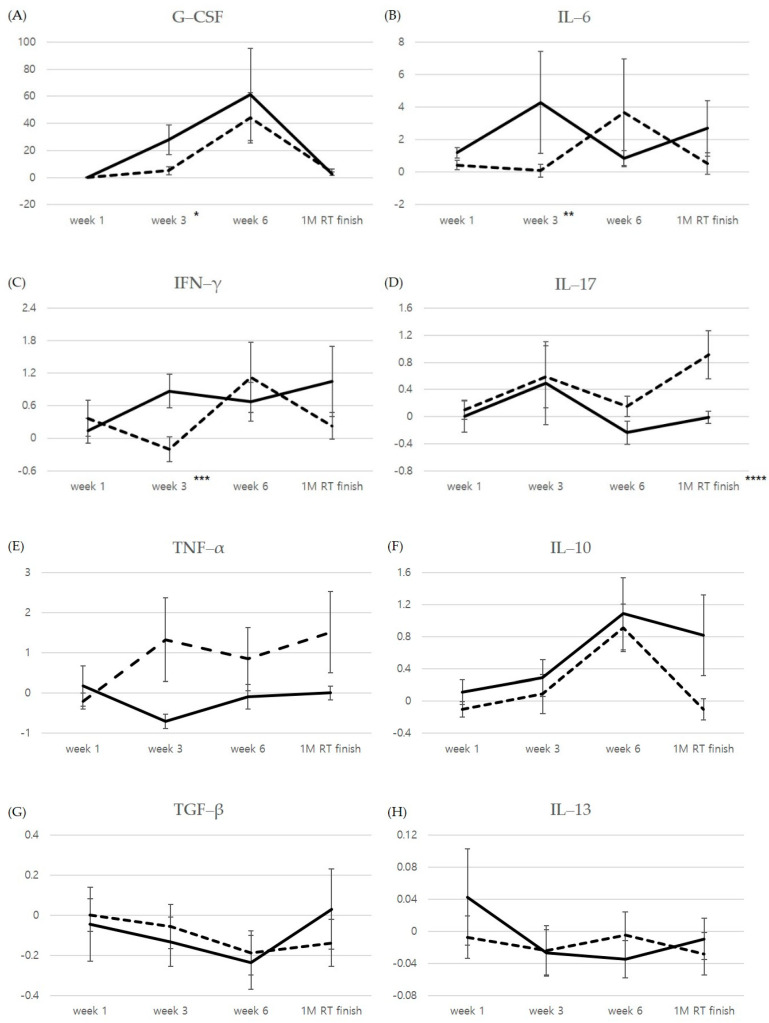
Relative changes in cytokine levels compared to pre-RT levels: (**A**) G-CSF; (**B**) IL-6; (**C**) INF-γ; (**D**) IL-17; (**E**) TNF-α; (**F**) IL-10; (**G**) TGF-β; (**H**) IL-13 levels. Data are expressed as the mean ± SE. Continuous line = Gr ≥ 2 radiation pneumonitis, Interrupted line = Gr 0–1 radiation pneumonitis. *p* = 0.026, * *p* = 0.050, ** *p* = 0.026, *** *p* = 0.045.

Figure 2 and Table 2 shows the relative changes compared to the pre-RT cytokine levels.

G-CSF levels continued to increase during RT, and the largest change compared to baseline occurred at 6 weeks in both groups. The differences between the 2 groups were significant at 3 weeks when compared to pre-RT levels (*p* = 0.026). IL-6 and IFN-γ increased at 3 weeks after RT initiation in the Gr ≥ 2 RP group, but decreased in the Gr 0–1 group. At 3 weeks after RT initiation, the relative changes compared to the pre-RT levels differed significantly between the 2 groups for IL6 and IFN-γ (*p* = 0.050, and 0.026, respectively).

At 1 month follow up after RT completion, the relative changes in IL-17 compared to baseline were significantly different between the 2 groups (*p* = 0.045). For TNF-α, IL-10, IL-13, and TGF-β, there was no time point at which the degree of relative change compared to the baseline significantly differed between 2 groups.

## 4. Discussion

Several studies have evaluated the cytokine levels related to lung damage secondary to radiation exposure. The evaluation timing and patients’ characteristics were diverse, and the significant findings reported were inconclusive [9,10,11]. Therefore, it has been difficult to apply a consistent criterion for cytokines as predictive factors for RP. This study aimed to identify relevant cytokines related to RP by evaluating blood samples obtained during CCRT of NSCLC patients. Only a few of the cytokines studied were associated with Gr ≥ 2 RP, and the degree of their change at a particular time point was important. G-CSF, IL-6, INF-γ, and their changes at 3 weeks after RT initiation were significantly predictive for Gr ≥ 2 RP.

One prospective study investigated the variation in IL-6, IL-10, and TNF-α levels in patients treated with RT for NSCLC [12]. They grouped the patients as Gr 0 or Gr 1–5 RP, and circulating cytokine levels were analyzed at baseline, every 2 weeks during RT, and at the end of RT. In their study, changes in IL-6 and IL-10 levels after 2 weeks of RT were significantly higher in those showing RP and in patients without RP, respectively. They suggested that the early change in IL-6 and IL-10, especially the opposite variation of these cytokines, may be relevant as predictive factors for RP. Our study also showed the importance of early changes in the levels of cytokines in predicting the occurrence and severity of RP. Although the timing of the analysis differed from that of the study by Arpin et al. [12], the relative changes in IL-6, and IFN-γ levels in patients showing Gr ≥ 2 RP was larger than that of the patients with Gr 0–1 RP at 3 weeks after treatment. Absolute values were not meaningful in predicting RP, and it was important to identify the degree of change at an appropriate time.

In the study by Arpin et al. [12], the patients were divided into no RP and RP groups. Gr 1 RP occurs approximately in 90% of the patients treated with RT; among them, only 20–30% of patients progressed to symptomatic RP. Therefore, it is important to identify patients who are likely to progress to Gr ≥ 2 RP after Gr 1 RP development. In this respect, we grouped patients as Gr 0–1 RP and Gr ≥ 2 RP. We also found that IL-6 is an important predictive factor for the development of symptomatic RP (Gr ≥ 2 RP).

Early changes in IL-6 levels were reported in another study analyzing cytokine levels among 12 NSCLC patients treated with RT or CCRT [13]. Changes in cytokine levels differed between patients treated with RT alone (*n* = 6 patients) and CCRT (*n* = 6 patients). Additionally, early changes in IL-6 levels was an important association factor, especially in patients with severe RP. More patients receiving CCRT were included in our study, and early changes in the IL-6 levels during radiation therapy were also confirmed to be important in the generation of severe RP.

IL-10 and IL-13 have been recognized to act as anti-inflammatory cytokines by inhibiting pro-inflammatory cytokine production. Their association with RP has been mentioned in a few papers, but the results are controversial [12,14]. In our study, the temporal changes in IL-10 and IL-13 were not significant, nor did they differ with the occurrence of Gr ≥ 2 RP. No time point showed a peak as compared to that of pro-inflammatory cytokines, which had a peak at 3 weeks after RT initiation. The change in anti-inflammatory cytokine levels were not considered to be of much help as a predictor of Gr ≥ 2 RP.

TGF-β is the most frequently mentioned cytokine, and results related to it have been reported variably [14,15]. In our study, TGF-β levels decreased continuously during RT, but the change was not significant. TGF-β is known to be secreted from lung tumors [6,16]; therefore, a decrease in TGF-β levels is considered to be associated with tumor mass reduction. It would be more important to evaluate TGF-β levels after RT completion than before RT initiation. In our patients, TGF-β decreased during RT in both groups, and at 1 month after RT completion, the TGF-β levels increased in patients with Gr ≥ 2 RP; however, the difference did not reach statistical significance. It is thought that estimating cytokine levels at more frequent intervals after RT completion will help identify patients with symptomatic RP.

G-CSF and INF-γ are not known to be significantly related to RP. Ao et al. investigated early cytokine changes in radiation-induced fibrosis sensitive (C57BL/6) and resistant mice (C3H) [11]. G-CSF and IL-6 had their peaks early after radiation exposure in the lung tissue, bronchoalveolar lavage (BAL) samples, and serum of radiation-induced fibrosis-sensitive mice. Furthermore, they showed correlations between cytokine levels in the tissue and the blood, suggesting the use of blood as a surrogate marker rather than lung tissue for predicting radiation toxicity. G-CSF is known as a regulatory cytokine that stimulates neutrophil production. Several studies have reported the development of pulmonary toxicity after G-CSF treatment [17], and it was reported that high neutrophil to lymphocyte ratios at radiologic RP was related to the progression to symptomatic RP [4]. It is believed that neutrophils activated by G-CSF may cause pulmonary toxicity by attacking alveolar capillary walls. Aso et al. analyzed the cytokine profiles of the BAL of 16 NSCLC patients treated with CCRT [18]. IFN-γ was overexpressed in patients with Gr 3–5 RP as compared to patients with Gr 1–2 RP, before RT initiation and 3 weeks after RT initiation. In our study, G-CSF and IFN-γ had a peak at 3 weeks after RT initiation in the Gr ≥ 2 RP group, and the change in the level of this cytokine between baseline and 3 weeks after RT initiation was also significantly related to the development of Gr ≥ 2 RP. In this regard, analyzing the early changes of G-CSF and IFN-γ is considered to help predict the severe RP.

Immunotherapy for NSCLC patients has been studied extensively in recent years [19,20]. Durvalumab consolidation therapy after CCRT has been recommended as standard treatment since the phase 3 PACIFIC trial, which showed benefits in PFS and overall survival with durvalumab compared to CCRT alone [7]. Recently, several studies compared the toxicity of consolidation therapy to that of CCRT alone. RP occurred more frequently in patients treated with CCRT followed by durvalumab vs. CCRT alone; the onset was 1–2 months later in the durvalumab group. Furthermore, severe RP occurred more frequently in the durvalumab group [21]. Schoenfeld et al. reported on a severe case of RP, and identified changes in circulating cytokines associated with RP after CCRT followed by immunotherapy [22]. There is little research on the cytokine changes that occur after immunotherapy; studies are warranted for evaluating the cytokines related to an increased risk of RP after immunotherapy.

Cytokine changes related to normal organ toxicities have been evaluated in other cancers. Christensen et al. [23] evaluated serum cytokine changes in 42 patients with prostate cancer treated with RT in relation to gastrointestinal toxicity. IFN-γ and IL-6 increased significantly during IMRT, and the increased expression of IL-2 and IL-1 was related to acute gastrointestinal and genitourinary toxicity. In another study, circulating cytokine levels were assessed before RT, after 1, 3 and, 5 weeks, at RT completion, and 1 month after completion [24]. This study showed that IL-6 was significantly associated with the severity of acute genitourinary and gastrointestinal toxicity. The authors suggested that in prostate cancer, cytokine changes during RT had a predictive role in gastrointestinal and genitourinary toxicity. Ng et al. [25] assessed the changes of pro-inflammatory cytokine receptors in patients with hepatocellular carcinoma treated with stereotactic radiotherapy. The patients who developed chronic liver toxicity showed significantly higher levels of sTNFRII, and lower levels of sCD40L and CXCL1 early during stereotactic body radiation therapy (SBRT); in addition, early death after SBRT was significantly related to high levels of sTNFRII and sIL-6R after one to two fractions of SBRT. In view of these findings, analysis of cytokine changes during radiotherapy may help identify patients with a high probability of normal organ toxicity.

This study has some limitations. It had a retrospective design, and also included a relatively small number of patients. There were missing data at the time of analysis; this is because blood collection was not planned prospectively, but was obtained by following up the patient before, during, and after radiation therapy. Combined analysis of cytokine levels and dosimetric factors has been suggested to improve the prediction of symptomatic RP [15]. However, the study used blood samples obtained from the human biobank, and could not access patients’ personal information; only the clinical information provided by the biobank could be obtained. Unfortunately, information on dosimetric factors cannot be obtained without access to the patients’ personal information and is against the recommendations of the IRB. Therefore, it was difficult to analyze the relationship between dosimetric factors and radiation pneumonitis in this study. However, as the organization complied with the dose constraint guidelines presented in the methods section, it is unlikely that the effects of dosimetric factors will be significant.

## 5. Conclusions

The cytokine levels studied showed various patterns of change, and these patterns varied depending on RP severity. The data showed that the evaluation of the changes in IL-6, G-CSF, and IFN-γ levels at 3 weeks after RT initiation would be of help to distinguish severe RP. The mechanisms for the various cytokines involved in RP severity warrant further investigation.

## Figures and Tables

**Table 1:** Clinical characteristics of patients with Grade ≥ 2 and Grade 0–1 radiation pneumonitis.

Characteristics		All Patients (*n* = 15)	Gr ≥ 2 (*n* = 6)	Gr 0–1 (*n* = 9)	*p*-Value
Sex	male	13	5	8	1.0
	female	2	1	1	
Age	years, median (range)	70 (54–76)	71.5 (58–75)	60 (54–76)	0.456
ECOG	0	3	0	3	0.229
	1	12	6	6	
Smoking history	none	4	1	3	0.604
	yes	11	5	6	
Underlying lung disease	none	5	1	4	0.580
	yes	10	5	5	
Clinical stage (7th edition)	IIIA	6	1	5	0.287
	IIIB	9	5	4	
Histology	SqCC	11	5	6	0.604
	adenocarcinoma	4	1	3	
Chemotherapy regimen	Paclitaxel-cisplatin	8	4	4	0.608
	Paclitaxel-carboplatin	7	2	5	
Total RT dose	Gy, median (range)	59.4 (55.8–66)	59.4 (59.4–66)	63 (54.8–66)	0.388
RT technique	3D CRT	7	1	6	0.119
	IMRT	8	5	3	

Abbreviations: ECOG = Eastern Cooperative Oncology Group; SqCC, = squamous cell carcinoma; RT = radiotherapy; 3D CRT = three-dimensional conformal radiotherapy; IMRT = intensity modulated radiotherapy.

**Table 2:** Relative changes in cytokine levels compared to pre-RT levels.

	Gr ≥ 2		Gr 0–1		*p* Value
	Mean	SE	Mean	SE	
G-CSF					
Week1	−0.035	0.227	−0.018	0.361	0.456
Week3	27.732	10.849	5.077	3.020	0.026
Week6	61.257	34.150	44.268	18.680	0.607
1M RT F	2.801	1.157	4.064	2.341	0.529
IL-6					
Week1	1.196	0.32	0.434	0.29	0.145
Week3	4.276	3.14	0.089	0.39	0.050
Week6	0.834	0.49	3.679	3.30	0.864
1M RT F	2.696	1.71	0.528	0.65	0.147
IFN-γ					
Week1	0.138	0.23	0.366	0.33	0.776
Week3	0.872	0.31	−0.203	0.23	0.026
Week6	0.674	0.36	1.120	0.65	0.955
1M RT F	1.048	0.65	0.226	0.25	0.438
IL-17					
Week1	0.002	0.23	0.099	0.14	0.607
Week3	0.491	0.61	0.586	0.46	0.414
Week6	−0.238	0.17	0.147	0.15	0.088
1M RT F	−0.010	0.09	0.916	0.35	0.045

Abbreviations: Gr = grade; RT = radiotherapy; M = month; F = finish. G-CSF= granulocyte colony-stimulating factor; IL=interleukin; IFN=interferon.

## Data Availability

The data presented in this study are available on request from the corresponding author.
